# A new click beetle genus from the Chilean Central Andes: Bohartina (Coleoptera, Elateridae, Elaterinae)

**DOI:** 10.1673/2006_06_31.1

**Published:** 2006-10-23

**Authors:** Elizabeth T. Arias

**Affiliations:** Essig Museum of Entomology, University of California, Berkeley, 210 Wellman Hall. Berkeley, CA 94720

**Keywords:** Bohartina vilchesensis, Bohartina palmae

## Abstract

Bohartina Arias, a new genus of Elateridae from forests in the Andean Cordillera of Central Chile, is here described and illustrated with 2 species: B. vilchesensis sp. nov. and B. palmae sp. nov. The genus Bohartina belongs to the subfamily Elaterinae and to the tribe Agriotini.

## Resumen

Se describe e ilustra Bohartina Arias, género nuevo de Elateridae de los bosques de la Cordillera Central de los Andes, con dos especies: B. vilchesensis sp. nov. y B. palmae sp. nov. El género Bohartina pertenece a la subfamilia Elaterinae y a la tribu Agriotini.

## Introduction

Chilean temperate forests still harbor an unknown number of undescribed arthropod taxa due to the current lack of intensive surveys and taxonomic work. In Chile, the family Elateridae includes 47 genera and 122 species (Arias 2005, 2004, 2001a, 2001b; Elgueta and Arriagada 1989; Blackwelder 1944). Click beetle specimens belonging to a new taxa were found among specimens of the genus Alyma Arias.

According to the classification of Gur’yeva (1974), Platia (1994) and Calder (1996), Bohartina belongs to the subfamily Elaterinae because it exhibits the following characters: head distinct, convex, narrow anteriorly; incomplete frontal carina across front of frons (although some subfamily members have a complete carina across front of frons); mesocoxae open to both mesepimeron and mespisternum; and tarsal claws without a basal setae on the outer flat portion. The genus  Bohartina, belongs to the tribe Agriotini because it exhibits the following characters: simple claws, third tarsal joint ventrally simple, and pronotosternal–pleural sutures furrowed at anterior ends (Platia 1994). The genus Bohartina is closely related to the genus Agriotes Eschscholtz. The genus Agriotes differs from the new genus Bohartina in the following characters: frontal antennal carina directed towards dorsal margin of labrum; prothorax parallel-side over the antennae which meets the dorsal labrum area, and female genitalia with enlarged colleterial glands (Becker 1958).

In Chile, Agriotini is represented by the genus Agriotes with five species: Agriotes australis Fairmaire 1883, A. chilensis Schwartz 1902, A. dubia Fleutiaux 1907, A. germaini Fleutiaux 1907 and A. vicinus Fleutiaux 1907. Agriotes australis and A. vicinus have been cited for Argentina (Golbach 1994). The new genus Bohartina (Elaterinae Agriotini) is proposed here with the following species: Bohartina vilchesensis Arias sp. nov. and B. palmae Arias sp. nov.

## Materials and Methods

Measurements were made with a calibrated ocular micrometer as follows: total body length (mm) from the frontal margin to apex of elytra and elytral width; the maximum width of the elytra, when both sides are in focus. Indices are indicated as follows: Pronotal elytral index [PEI] is obtained by dividing the length of the pronotum by the length of the elytra (Calder 1996). The pronotal elytral index is used here because it gives a general idea in how big the pronotum is compared with the elytra. The pronotal index [PI] is obtained by dividing the length of the pronotum by its width. Elytral index [EI] is obtained by dividing the length of the elytron by its width. Antennomere proportion [AP] is the lengths of antennomeres 2 through 11 as 1/100^th^ of the total antennal length. This is not measured for antennomere 1 because it is curved and hard to measure. Body length is measured from dorsal view including the head. Tarsomere proportion [TP] gives the lengths of the tarsomeres as 1/100^th^ of the total tarsal length.

Specimens from which the genitalia were to be removed were first relaxed overnight in warm water with a few drops of soap added. For examination of male genitalia, the last abdominal segment was removed and placed in water with a few drops of soap in a Petri dish and left over night. Then, genitalia were removed and glued to a point card on its lateral side with balsam, and placed on the pin under the specimen.Becker (1958) was followed for female genitalia examination.

Drawings were made using a camera lucida on a dissecting scope Leica MZ7. All dates in the records given were converted to a standard format of day.month.year, with the month given in Roman numerals. Places and names in the recorded labels are the original spellings.

Museums and institutions that contributed to this work are indicated in the acknowledgements and, in the text, by the acronyms in brackets (Arnett et al 1997), excluding [ETA] author’s collection. Type specimen repositories are also indicated in descriptios.

## Taxonomy

###  Bohartina Arias Gen. Nov. (Figs. 1,2, 3)

#### Type species

 Bohartina vilchesensis Arias, sp. nov., here designated.

#### Description

**Figure 1:**
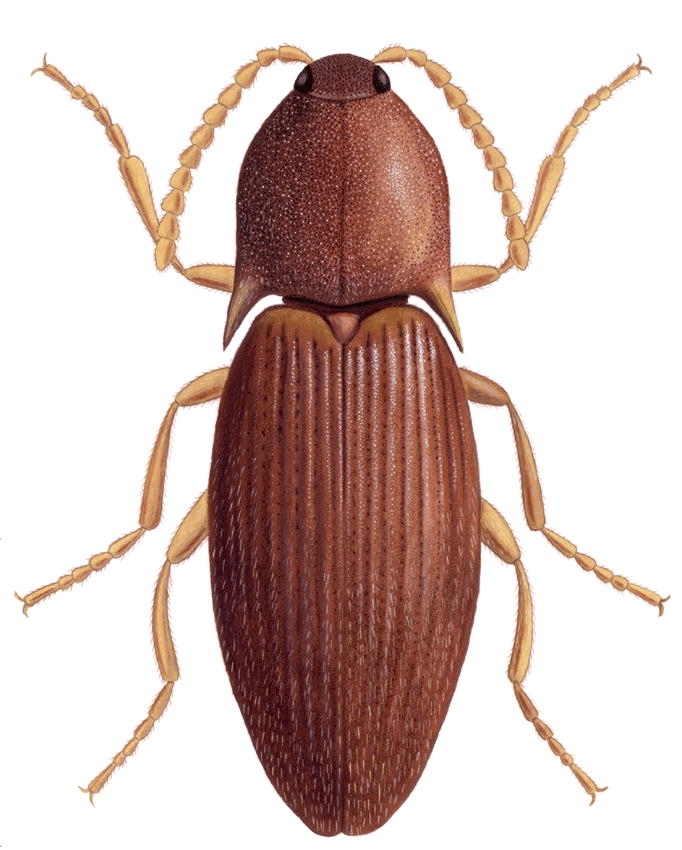
Dorsal habitus of Bohartina vilchesensis. Illustration Nancy V. Arias T.

**Figures 2–3:**
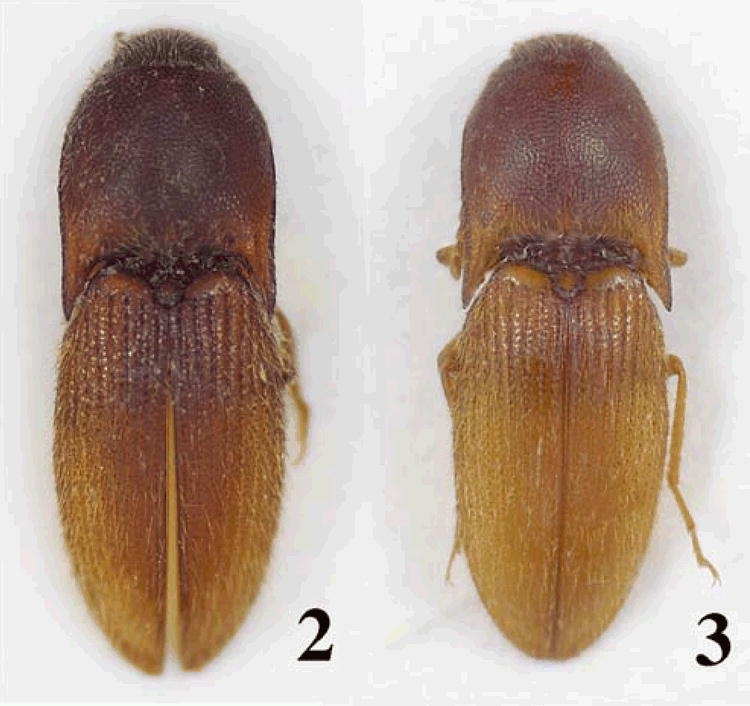
Photos of Bohartina species: B. vilchesensis sp. nov. 2, B. palmae sp. nov. 3.

Body stout (Figs. 1,2, and 3)Color brown, red brown, red yellow, light brown; integument semi-shiny or dull; length 4.81–5.26 mm, width 1.54–1.82 mm.

**Figure 4:**
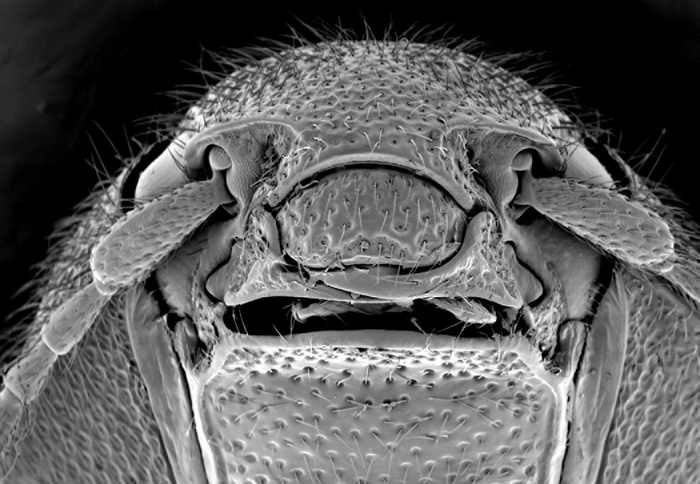
Scanning electron micrograph of head of Bohartina vilchesensis.

##### Head

Declivous, punctate; vestiture long, yellow or gold, semi-erect, or semi- decumbent, sparse or dense; frontoclypeal region sloping to base of clypeus; frontal carina incomplete across front of frons; eyes small, [EI: 0.25]; clypeus present; labrum exposed and rounded in shape; eleven antennomere not reaching apex of posterior pronotal angles; first and second antennomere conical, remaining antennomeres serrate, with dense vestiture semi-erect; mandibles bidentate, maxillary and labial palps with apical segments securiform; antennal groove present, carinate in pronotosternal hypomeral side.

##### Prothorax

Convex; [PI: 0.78–0.96]; strongly narrowed anteriorly to receive head; lateral margins entirely carinate, sinuate, inclined mesodorsally, lateral carina directed ventrally, pronotal lateral margin not joining pronotosternal suture apex; pronotal punctures areolate, distinct; pronotal basal area strongly declivous to prescutum; pronotal basal margin curved; prescutum notch small, V-shaped; posterior angles long, acute, unicarinate, 0.35X pronotal length; prosternum as long as wide, convex; pronotosternal lobe bent; pronotosternal suture thickened giving a double appearance, furrowed at anterior end, curved at procoxal margin; articulation of prothoracic sternite around procoxae acute, directed outward, and marginate; pronotosternal hypomeron punctate; pronotosternal spine long and following procoxae, globular, marginate. Scutellum oval in shape; posterior margin of mesosternal cavity extending in distance posteriorly shortly, cavity deep; mesocoxae longer than wide; mesocoxal cavity deep, open to mesepimeron and mesepisternum; mesosternum and metasternum separated by distinct external suture. Elytra: parallel-sided medially; striate, striae deeply incised; apex truncate. Metathoracic wings not present in any individuals so far studied; metathoracic coxal plate widest region closest to medial body line; with setae semi-decumbent, gold. Leg: femur globate; tarsi 1 through 4 decreasing in length distally, tarsomere 4 very small in size compared with other tarsomera.

##### Abdomen

Punctate; last abdominal ventrite angulate.

##### Genitalia

Female: 1.97 mm wide. Vagina without sclerotized internal structures; strongly enlarged towards the apex, 3X base wide; bursa copulatrix globular, with two sclerotized fan shaped structures with teeth alternating between long and short and another long sclerotized structure dorsal; spermatheca consist of two delicate non-sclerotized spiral structure attached to bursaventrally; spermatheca gland at end of bursa (Fig. 3a, b). Male: with a sclerotized structure ventrally as a brush behind aedeagus (Fig. 11b). Female genitalia did not exhibit differences at generic level.

##### Distribution

Andes Cordillera and Coastal Cordillera, Region VII of Chile.

##### Biology

Adult specimens were collected during December. Species from this new genus did not have distinctive sexual dimorphism and specimens need to be dissected to discriminate between males and females.

##### Etymology

The designation of this genus is in honor of Richard M. Bohart for his great contribution and dedication to the teaching of systematics.

###  Bohartina vilchesensis sp. nov. Arias (Figs. 1, 2, 4, 6, 8, 10, 12)

#### Description

Body stout (Figs 1 and 2) Length 4.96 mm including head (head 0.41 mm), width 1.67 mm measured at widest point. Color dark brown; integument dull; vestiture semi-erect, pale. [PEI: 2.34–2.52].

##### Head

Supra antennal carina distinct (Fig. 4); labrum 1.66 X times as long as wide; antennomere 1–2 conical, remaining ones serrate; [AP: 9.59-7.07-10.60-10.60-8.58-10.10-11.11-9.59-10.10-12.66], (Fig. 6). Prothorax: areolate. [PI: 0.96]. Posterior angles not divergent; prosternum convex; pronotosternal hypomeron areolate; antennal groove carinate on pronotosternal hypomeral side as an impression, (Fig. 8); prosternum at procoxae marginate, procoxae separated by 0.52 X times procoxal diameter; pronotosternal spine 1.23 X procoxal diameter. Scutellum oval, 1.22 X times as long as wide; mesocoxae separated by 0.53 X times mesocoxal diameter; posterior margin of mesosternal cavity extending posteriorly 0.38 X times mesocoxal diameter. Elytra: punctate, punctures dense; elytral anterior border carinate and striate, vestiture dense; apex truncate. Leg: vestiture light yellowish; tarsomere III oblique, tarsomere IV oblique and small, [TP: 34:19:18:11:18].

**Figure 5:**
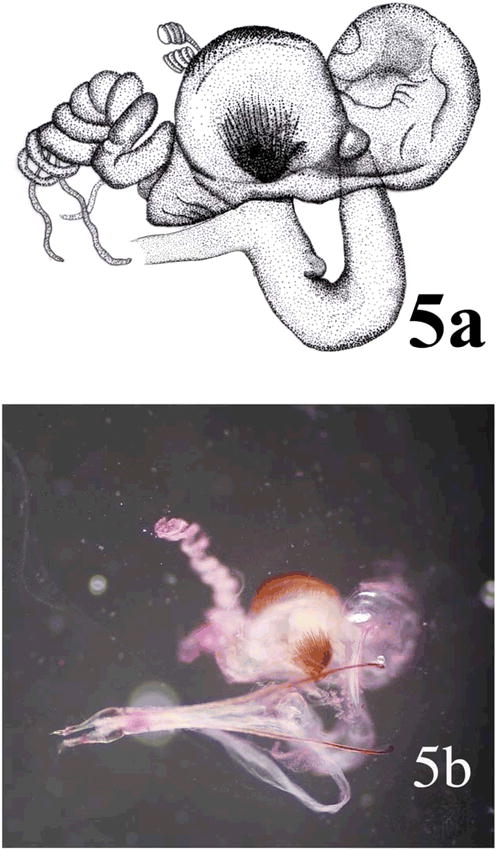
Female genitalia of Bohartina vilchesensis: drawing of a dorsal view, (a) and a photograph of a ventral view (b).Illustration by Nancy V. Arias T. (Scale bar =0.5 mm).

**Figures 6–7:**
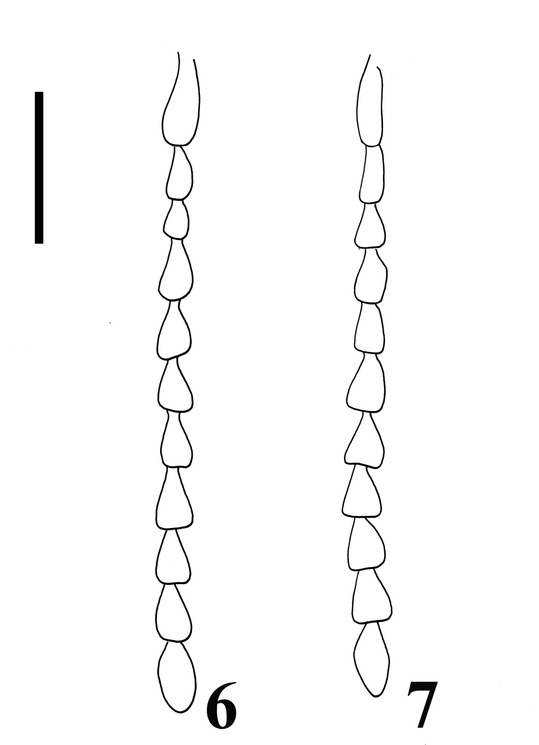
Antennae of Bohartina species, 6, B. vilchesensis; 7,  B. palmae (Scale bar =0.5 mm.).

**Figures 8–9:**
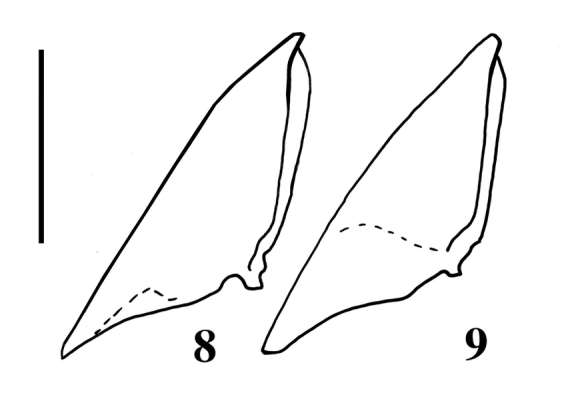
Pronotosternal hypomera of Bohartina species. 8, B. vilchesensis; 9, B. palmae. (Scale bar =1.0 mm).

##### Male genitalia

**Figures 10–11:**
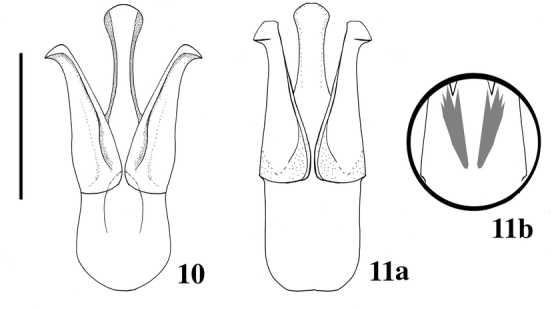
Male genitalia of Bohartina species. 10, B. vilchesensis. 11a, B. palmae. 11b, B. palmae ventral area of male genitalia outlining brush. (Scale bar =0.5 mm).

0.44 mm long, 0.22 mm wide (Fig 10).

##### Material Studied

Holotype. Male: 4.96 mm in length, 1.67 mm in width. “CHILE, VII, Talca, Altos de Vilches, December 1978. L. E. Peña”. [ETA]. Paratypes 6: 4 Males and 2 females: “CHILE, VII, Talca, Altos de Vilches, December 1978. L. E. Peña”. [ETA]. Other material studied: 2 males: Vilches Alto. Oct. 1990.

##### Biology

There is no other currently available information on the biology of this species.

##### Distribution

**Figure 12:**
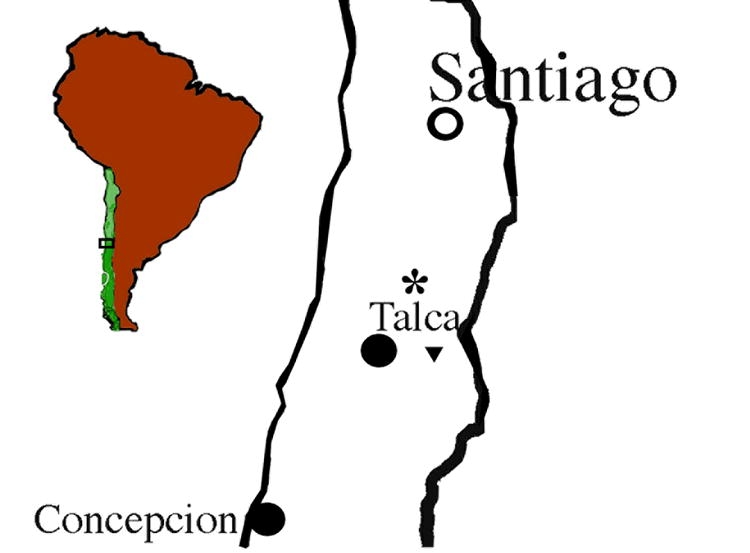
Distribution of species of Bohartina: B. vilchesensis, B. palmae.

Altos de Vilches, Talca, Cordillera of VII Region, Chile (Fig. 12).

##### Remarks

 Bohartina vilchesensis can be recognized by its stout appearance, dull tegument, dark brown color, dense pale vestiture, convex, anteriorly narrow pronotum and truncate elytral apex.

##### Etymology

This species name is after the type locality Vilches where it was collected.

###  Bohartina palmae Arias sp. nov. (Figs. 3,7, 9, 11a, 11b, 12)

#### Description

Body stout (Fig. 3) Length 4.29 mm including head (head 0.38 mm), width 1.69 mm at widest point; red brown; integument semi-shiny; vestiture semi-erect, gold. [PEI:2.52].

##### Head

Labrum 1.66 X as long as wide; antennomere 1–2 conical, remaining ones serrate, antennomere 11 does not reaches apex of posterior angles, [AP:10.76-8.20-9.74-8.71-10.76-9.74-9.23-9.74-9.23-13.92], (Fig. 7). Prothorax: distinctly areolate; posterior angles divergent; prosternum convex; hypomeron rugulose, antennal groove carinate on the pronotosternal humeral side as an impression, (Fig. 9); prosternum at procoxae marginate, procoxae separated by 0.52 X procoxal diameter; pronotosternal spine 1.26 X procoxal diameter. Scutellum: oval, 1.06 X as long as wide; mesocoxae separated by 2 X mesocoxal diameter; posterior margin of mesosternal cavity extending posteriorly 0.21 X mesocoxal diameter. Elytra: punctate, punctures dense; elytral striae with punctures and striate; vestiture dense, semi-erect, gold, lacking at suture area; apex distinctly truncate towards elytral suture. Leg: vestiture gold; tarsomere III oblique tarsomere IV small, [TP: 37:23:11:10:20].

##### Male genitalia

0.59 mm long, 0.24 mm wide, apex convex (Fig. 11a, 11b).

##### Material Studied

Holotype. Male. 4.29 mm in length. “CHILE Fundo Malcho Parral Cord Parral Dec. 1957”. Paratypes: females (n= 7). “CHILE Fundo Malcho Parral Cord Parral Dec. 1957”. L. E. Peña”. Paratypes: males (n=2). “CHILE, VII, Talca, Altos de Vilches, December 1978. L. E. Peña”. [ETA].

##### Other material studied

Females (n= 7). “CHILE Fundo Malcho Parral Cord Parral Dec. 1957. L. E. Peña”.

##### Biology

There is no other currently available information on the biology of this species.

##### Distribution

Parral and Talca, Cordillera of VII Region, Chile (Fig. 12).

##### Remarks

 Bohartina palmae can be recognized by its stout appearance, shiny brown reddish integument, spare gold vestiture, and strongly convex and anteriorly narrow pronotum.

##### Etymology

This species name honors Palma Lower (University of California Davis) for her enthusiasm and encouragement towards my beetle research.

## Discussion

All the specimens belonging to the genus Bohartina, used in this study, lack methatoracic wings. This trait, absence of methatoracic wings, has been found in Coleoptera before. The Chilean genus Alyma Arias also has the same character (absence of methatoracic wings). It is presently unknown if this trait is characteristic of both taxa. Phylogenetic studies of the genera that belong to the tribes Pomachiliini and Agriotini are yet to be completed.

## Abbreviations

PEI: pronotal elytral index
PI: pronotal index
EI: elytral index
AP: antennomere proportion
TP: tarsomere proportion
